# Case Report: High TMB fails to predict immunotherapy response in *PTCH1*-mutant basal cell carcinoma with squamous differentiation

**DOI:** 10.3389/fimmu.2026.1814345

**Published:** 2026-09-22

**Authors:** Esranur Aydın, Ünal Metin Tokat, Ashkan Adibi, Şevval Nur Bilgiç, Eylül Özgü, Alina Melisa Manea, Mutlu Demiray

**Affiliations:** Precision Oncology Center, Medicana Health Group, Istanbul, Türkiye

**Keywords:** basal cell carcinoma (BCC), UV mutational signature, hedgehog signaling pathway, immunotherapy resistance, precision oncology, *PTCH1*, squamous cell carcinoma (SCC), tumor mutational burden (TMB)

## Abstract

**Background:**

Tumor mutational burden (TMB) is widely utilized as a predictive biomarker for response to immune checkpoint inhibitors (ICIs). However, a subset of hypermutated tumors exhibits primary resistance to immunotherapy, indicating that additional tumor-intrinsic mechanisms may override immunogenicity. Aberrant Hedgehog (Hh) signaling, frequently driven by *PTCH1* inactivation in basal cell carcinoma (BCC), has been implicated in immune exclusion and suppression of antitumor immunity.

**Case presentation:**

We report a case of a 64-year-old male initially diagnosed with cutaneous squamous cell carcinoma (SCC). Comprehensive genomic profiling (CGP) of archival tumor tissue revealed an ultra-high TMB (95 muts/Mb) accompanied by a distinct ultraviolet (UV) mutational signature, with negative PD-L1 status. Based on the elevated TMB, dual immune checkpoint blockade was initiated; however, the patient did not achieve objective tumor regression. Reassessment of the genomic data, specifically the presence of a pathogenic truncating *PTCH1* (Y977fs*16) alteration alongside the UV signature, raised a strong suspicion of basal cell carcinoma with squamous differentiation, prompting a diagnostic revision. Given the established role of PTCH1 loss in constitutive Hedgehog (Hh) pathway activation and immune exclusion, we considered aberrant Hedgehog signaling to be a biologically plausible contributor to the limited objective response observed during initial immune checkpoint blockade. Consequently, a rational combinatorial strategy utilizing vismodegib (an Hh pathway inhibitor) and pembrolizumab was initiated.

**Results:**

Following the introduction of the targeted-immunotherapy combination, the patient achieved a durable and ongoing clinical response. Serial liquid biopsy analysis showed that the same PTCH1 alteration remained detectable during longitudinal assessment, supporting the biological relevance of Hedgehog pathway dysregulation in this case.

**Conclusion:**

This case highlights the limitations of relying solely on conventional biomarkers, such as TMB, even in the context of extreme hypermutation and UV-induced mutagenesis. CGP may be essential not only for therapeutic guidance but also diagnostic refinement. Our findings support the hypothesis that Hedgehog pathway inhibition may contribute to overcoming apparent resistance to immune checkpoint blockade in selected PTCH1-altered BCCs, supporting a biology-guided combinatorial approach in biomarker-discordant tumors with high TMB.

## Introduction

Cutaneous squamous cell carcinoma (cSCC) and basal cell carcinoma (BCC) represent the most common non-melanoma skin cancers. While most cases follow an indolent course, a subset of patients develop locally advanced or metastatic disease, which is associated with limited therapeutic options and poor outcomes (1). In such settings, immune checkpoint inhibitors (ICIs) have emerged as an important treatment modality; however, treatment responses remain heterogeneous and challenging to predict, particularly in tumors with overlapping histopathological features or divergent biological behaviors (2).

Programmed death-ligand 1 (PD-L1) expression and tumor mutational burden (TMB) are among the most widely used biomarkers for guiding immunotherapy decisions in solid tumors (3). High TMB is generally correlated with an increased neoantigen load and improved response to ICIs, especially in tumors with ultraviolet (UV)-related mutagenesis (4). Nevertheless, clinical experience has demonstrated that a high TMB alone does not uniformly translate into durable benefit from immunotherapy (5). This highlights the limitations of relying on single biomarkers and underscoring the necessity for a more integrated understanding of tumor biology (6).

Emerging evidence suggests that tumor-intrinsic oncogenic signaling pathways may influence the tumor microenvironment and modulate sensitivity to immune checkpoint blockade. Hedgehog/GLI signaling has been associated with changes in stromal organization and immune-cell recruitment in preclinical and translational models, providing a biological rationale for pathway-directed combinational treatment strategies. (Grund-Gröschke, Stockmaier and Aberger, 2019; 7).

Molecular tumor boards (MTBs) integrating CGP have become central to identifying actionable alterations and guiding treatment strategies across diverse solid tumors, as demonstrated in multiple cases showing durable responses driven by genomics-guided therapy decisions (8–10).Here, we report a case evaluated in a MTB in which the discordance between conventional immunotherapy biomarkers and clinical response prompted a comprehensive re-evaluation of tumor biology. The integration of longitudinal histopathological, immunohistochemical, and molecular findings led to a diagnostic revision and informed a rational combination of immune checkpoint blockade with Hedgehog pathway inhibition, resulting in a sustained, near-complete response. This case highlights the importance of moving beyond conventional biomarkers and illustrates how integrated molecular and pathological assessments can optimize clinical decision-making in immuno-oncology.

## Case presentation

A 64-year-old male patient was initially diagnosed in 2021 with cutaneous squamous cell carcinoma located on the nasal dorsum. Following surgical excision in September 2021 and adjuvant radiotherapy, imaging performed in early 2022 revealed metastatic disease involving the dens of the C2 vertebra and the posterior half of the C7 vertebral body. At the time of metastatic presentation in September 2021, the patient reported severe neck pain and marked restriction of cervical range of motion without preceding trauma. Physical examination revealed cervical percussion tenderness and muscle spasm, with painful restriction of neck movements, but no neurological deficit. His performance status was 1, and he was using a cervical collar. PET/CT showed multiple lytic/hypermetabolic osseous lesions involving the axial skeleton, pelvis, and sternum, without documented visceral involvement at that time. Histopathological evaluation of the metastatic lesion at that time confirmed the cSCC metastasis. The patient subsequently received carboplatin plus paclitaxel, with carboplatin dose reduction during treatment due to emesis, and later zoledronic acid was added after dental evaluation. He also recieved radiotherapy targeting the metastatic sites.

In June 2022, the patient presented to our clinic for a second opinion. To reassess the extent of the disease, a baseline PET-CT scan was performed, and CGP was requested from the archived surgical specimen (September 2021). Tissue CGP was performed using the FoundationOne CDx assay (full coding exon coverage for all 324 genes; select intronic regions in 36 genes for rearrangement detection; FFPE archival tissue). PD-L1 IHC was performed in parallel by Histogenomics GbR (Ventana SP263). The CGP results (July 2022) revealed a PD-L1 negative tumor (Combined Positive Score (CPS): 0, Tumor Proportion Score (TPS): 0%) harboring an ultra-high TMB of 95 muts/Mb. The pathogenic genomic alterations were identified in *BRCA1, PTCH1, ATM, ASXL1, ATR, CUL3, EZH2, HGF, MLL2, SNCAIP, TERT*, and *TP53*. The results of tissue- and liquid biopsy-based comprehensive genomic profiling are summarised in **Table 1**.

**Table 1:** Summary of comprehensive genomic profiling results.

Parameter	Tissue CGP (06 July 2022)	Liquid biopsy CGP (19 January 2023)
Sample source	Primary tumor (nasal lesion, surgery 09/23/2021)	Peripheral blood (sample taken 04 January 2023)
Microsatellite status	MS-Stable	MS-Stable
PD-L1 expression	CPS: 0, TPS: 0%	Not assessed
Tumor mutational burden	95 mutations/Mb	85 mutations/Mb
Genomic alterations	***BRCA1*** Q1756fs*74;* ***ATM*** *R1898*; ***PTCH1*** Y977fs*16;* ***ASXL1*** *R417*; ***ATR*** R2363*; ***CUL3*** Q664*; ***EZH2*** W60*; ***HGF*** W415*; ***MLL2*** Q4364*; ***SNCAIP*** P734S; ***TERT*** promoter −146C>T; ***TP53*** E286K, T211I	***BRCA1*** Q1756fs*74;* ***ATM*** *R1898*; ***PTCH1*** Y977fs*16;* ***ASXL1*** *R417*; ***ATR*** R2363*; ***CUL3*** Q664*; ***EZH2*** W60*; ***HGF*** W415*; ***MLL2*** Q4364*; ***SNCAIP*** P734S; ***TERT*** promoter −146C>T; ***TP53*** E286K, T211I; ***TET2*** splice site 3595-2A>G, Q138*; ***SMAD4*** Y195*

Bold values represent standard gene symbols/names.

Despite the lack of PD-L1 expression, the markedly elevated TMB provided a strong rationale for immunotherapy. Combined immune checkpoint blockade was initiated with nivolumab 400 mg every 4 weeks and ipilimumab 50 mg every 8 weeks. Given the absence of PD-L1 expression and the presence of an ultra-high tumor mutational burden, a dual checkpoint strategy was selected with the aim of maximizing immune activation in a patient with rapidly progressive metastatic disease. However, during follow-up evaluations, the patient failed to achieve the clinical and radiological responses typically anticipated for an ultra-high TMB tumor, achieving only stable disease, as shown in **Figure 1**.

**Figure 1:**
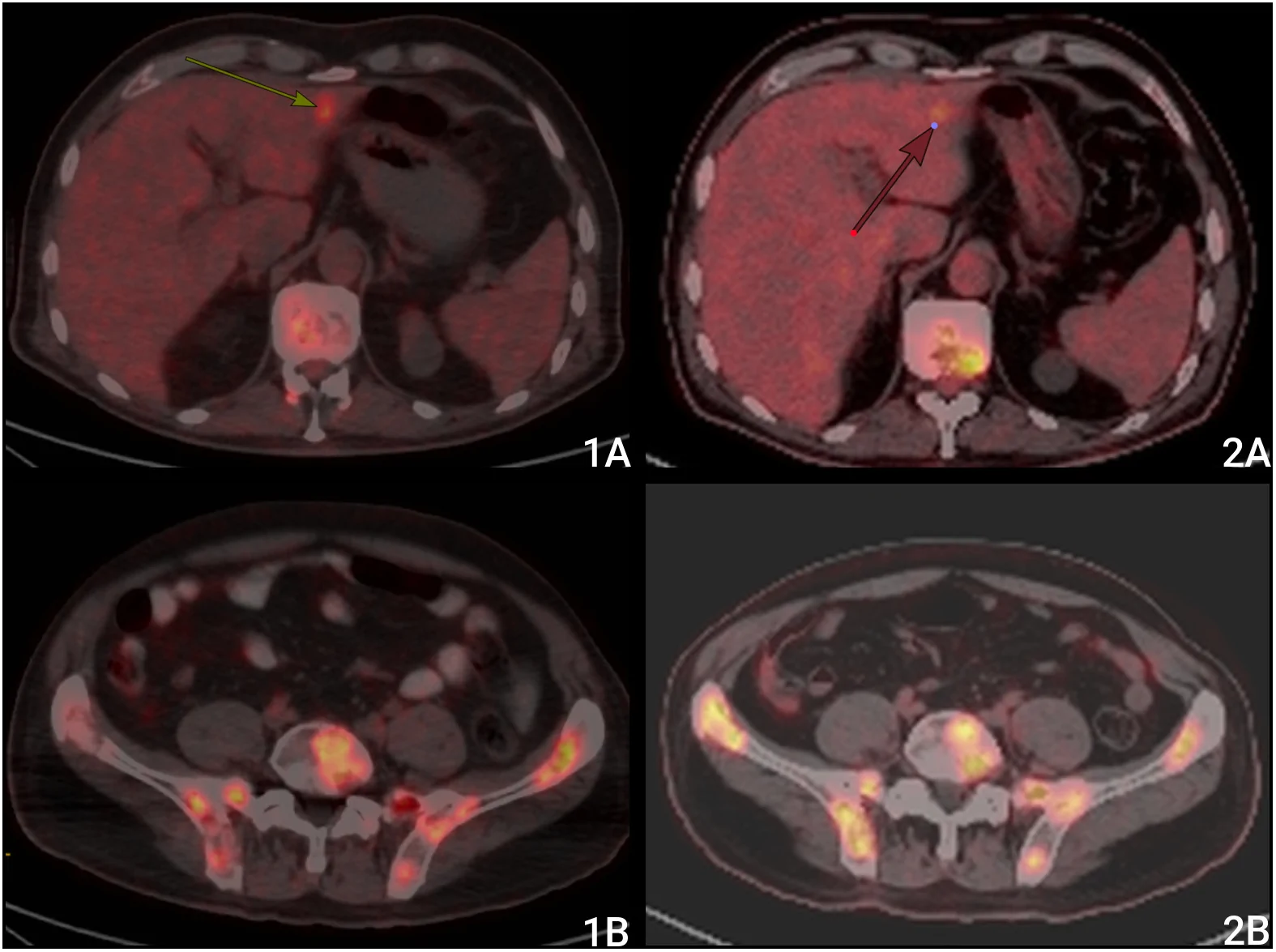
**(a)** June 2022: Baseline PET-CT scan demonstrating a hypermetabolic metastatic nodular lesion in segment III of the left hepatic lobe (arrow), **(1B)** diffuse, intense hypermetabolic metastatic foci throughout the iliac bones and vertebral *column.* December 2022 **(2A)** Follow-up imaging demonstrates stable metabolic activity in the previously identified hepatic metastatic lesion (arrow), without a significant interval change. **(2B)** The metastatic lesions involving the bilateral iliac bones and vertebral column similarly show no significant change in metabolic activity compared with the baseline examination.

To further investigate the potential mechanisms underlying the limited response and assess tumor heterogeneity and clonal evolution, liquid biopsy-based CGP analysis was performed in January 2023. CGP was performed using the FoundationOne Liquid CDx assay which interrogates 324 genes. This panel evaluates 311 genes for substitutions and indels, 310 genes for copy number alterations, all 324 for rearrangements from peripheral blood’s cfDNA. This analysis confirmed the persistence of previously identified genomic alterations and revealed newly emerged alterations in *TET2* and *SMAD4*. TET2 and ASXL1 alterations were interpreted cautiously because matched leukocyte sequencing was not performed and a contribution from clonal hematopoiesis could not be excluded. The TMB remained high at 85 muts/Mb.

Concurrently, the tumor’s ultraviolet (UV) mutational signature, together with the presence of a truncating *PTCH1* alteration, prompted a collaborative retrospective review with a pathologist. Given that the diagnostic evaluations and pathological assessments of the patient were performed at different time points and across multiple centers, all available histopathological, immunohistochemical, and molecular data were retrospectively reviewed and integrated. This review of primary nasal tumors demonstrated a dermal-based infiltrative growth pattern composed of basaloid tumor islands with areas of squamous differentiation and associated desmoplastic stroma. Immunohistochemical analysis revealed BER-EP4 positivity, diffuse nuclear p63 expression, and focal EMA positivity. Taken together, these findings indicated that the tumor biology was more consistent with metastatic cutaneous basal cell carcinoma with squamous differentiation rather than conventional cutaneous squamous cell carcinoma. Following a multidisciplinary discussion and further pathological review, the diagnosis was revised accordingly.

Dedicated germline testing of peripheral-blood constitutional DNA performed independently at an external local laboratory reported the BRCA1 Q1756fs*74 alteration as a germline variant. The patient was referred to another clinic for genetic counseling.

Given the presence of the *PTCH1* alteration in Hh pathway hyperactivation and its potential to mediate resistance to immunotherapy, the treatment strategy was shifted. The patient was transitioned to a rational combination of pembrolizumab (200 mg every 3 weeks) and the Hedgehog pathway inhibitor vismodegib (150 mg once daily), which was initiated concurrently. Pembrolizumab and vismodegib were initiated concurrently on the basis of the revised diagnosis, the established therapeutic role of Hedgehog-pathway inhibition in advanced BCC, and the rationale for maintaining PD-1 blockade. Clinical experience with this approach remains limited, and existing evidence does not establish additive or synergistic efficacy. Follow-up imaging performed within 3–4 months demonstrated a near-complete response as can be seen in **Figure 2**. The patient maintained this response until June 2025, when the treatment was stopped due to grade 2 pneumonitis. Pembrolizumab and vismodegib were interrupted, and the patient was followed closely under the supervision of the treating pulmonologist. The pulmonologist elected close clinical observation without systemic corticosteroids following treatment interruption, and the respiratory symptoms subsequently resolved. Following symptom resolution, vismodegib monotherapy was reintroduced approximately one month later. Recurrence of respiratory symptoms prompted permanent discontinuation of systemic treatment. Although the temporal sequence influenced this clinical decision, definitive attribution to vismodegib was not possible. Due to the lack of disease progression on the subsequent PET-CT imaging and the patient’s request, therapy was ultimately discontinued, and the patient remained on active surveillance. Strikingly, a sustained response was confirmed on the most recent PET-CT scan performed in November 2025. The treatment course is summarised in **Table 2**, while serial FDG PET/CT findings and metabolic response over time are presented in **Table 3**.

**Figure 2:**
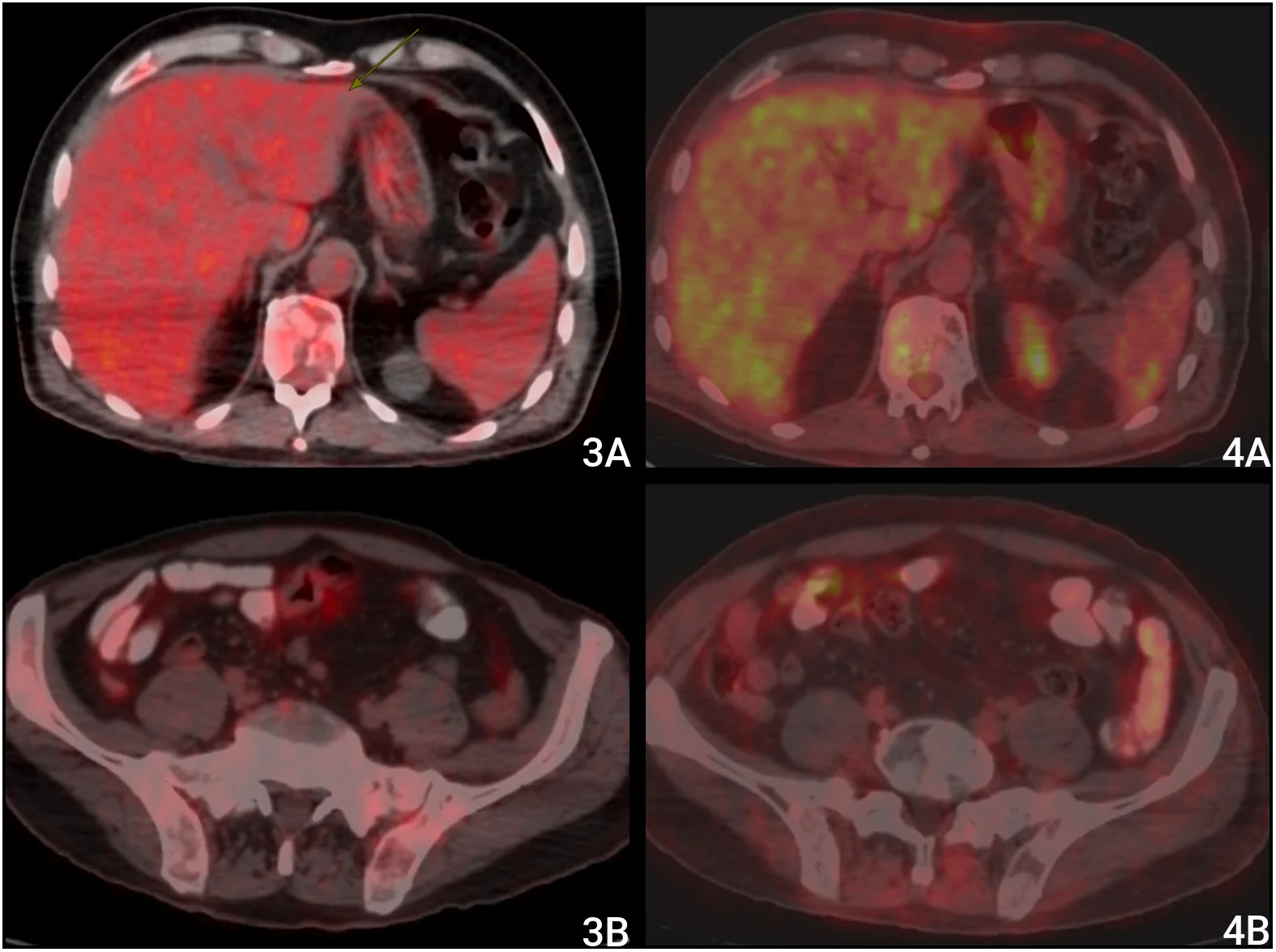
**(3A)** June 2023: PET-CT imaging reveals a complete metabolic response of the metastatic lesion in the segment III (arrow) of the left hepatic lobe. Partial metabolic regression is also observed in the metastatic areas of the iliac bones and vertebral column **(3B)**. **(4A)** November 2025: Long-term follow-up PET-CT scan confirming a sustained complete metabolic response in liver, **(4B)** Stable, metabolically inactive sclerotic metastatic lesions persisted in the iliac bones and vertebral column with no evidence of metabolic progression.

**Table 2:** Timeline of diagnostic reassessment and treatment course.

Time point	Key events	Molecular findings	Treatment	Clinical outcome
Sept 2021	Nasal tumor resection	Initial diagnosis: cSCC	Surgery + adjuvant RT	Local control
Early 2022	Bone metastases detected	—	Chemotherapy + palliative RT	Disease progression
June–July 2022	Second opinion; baseline PET-CT; tissue CGP	High TMB; PD-L1 negative; *PTCH1* mutant; UV signature	Nivolumab + Ipilimumab	Stable disease
Early 2023	Liquid biopsy CGP & Integrated pathology review	High TMB, persistent *PTCH1 mutation;* Revised diagnosis: BCC with squamous differentiation	Continued ICI	No meaningful regression - Diagnostic refinement
Mid 2023	Biology-guided strategy initiated	*PTCH1*-driven Hedgehog activation suspected	Pembrolizumab + Vismodegib	Rapid near-complete response
June 2025	Immune-related pneumonitis	—	Treatment discontinued	No radiologic progression
Nov 2025	Long-term follow-up PET-CT	—	Active surveillance	Sustained metabolic remission

**Table 3:** Serial FDG PET/CT comparison of representative metastatic lesions and metabolic response over time.

Representative lesion/site	June 2022 PET/CT	June 2023 PET/CT	November 2025 PET/CT	Overall metabolic response
Hepatic metastasis, segment III	Ill-defined hypodense nodular lesion measuring 13 mm in segment III of the left hepatic lobe with increased FDG uptake; S**UVmax 5.7**.	No pathological FDG uptake was detected within the hepatic parenchyma, consistent with a complete metabolic response of the hepatic lesion.	No pathological FDG uptake was detected within the hepatic parenchyma.	**Complete metabolic response achieved by June 2023 and sustained through November 2025**.
Axial and proximal appendicular skeleton	Multiple, widespread, intensely FDG-avid osseous metastatic lesions, some with lytic morphology; representative SUVmax: **10.8.**	Marked interval metabolic regression of osseous lesions; representative, SUVmax decreased **to 4.3**, corresponding to an approximately **60% reduction**.	Stable sclerotic metastatic lesions in the iliac bones and vertebral column without pathological FDG uptake, consistent with metabolically inactive treated lesions.	**Partial metabolic response of osseous disease by June 2023, followed by complete metabolic response/metabolic inactivity of residual sclerotic lesions by November 2025**.

Bold values represent key quantitative values and notable responses.

## Discussion

The FDA-approved tumor-agnostic threshold for ICI eligibility is ≥10 muts/Mb, while UV-driven skin cancers commonly exceed 50 muts/Mb. In cSCC, TMB values above 10–20 muts/Mb have been associated with improved ICI outcomes, whereas the predictive significance of TMB in BCC is less well established and may be confounded by the immunosuppressive character of the BCC TME (2, 11).

This case illustrates a clinically meaningful discordance between TMB and immunotherapy responsiveness. The tumor displayed an ultra-high TMB (95 mutations/Mb) together with a dominant ultraviolet (UV) mutational signature, features commonly associated with increased neoantigen load and improved ICI outcomes in multiple cancer types (11–14). However, the absence of PD-L1 expression and the stable disease without objective regression from combined nivolumab and ipilimumab underscore the complexity of biomarker interpretation. While high TMB can enrich for responders, it does not function as a context-independent predictor of immune sensitivity (2, 5, 15). Similarly, a comprehensive review of predictive biomarkers in non-melanoma skin cancers highlighted that, despite the high mutational burden of BCC and cSCC, validated tumor-specific predictors of immune checkpoint inhibitor response remain limited, underscoring the need to interpret TMB together with the broader tumor–immune context (16). In this patient, ultra-high mutational burden was not accompanied by objective tumor regression during initial checkpoint blockade, suggesting that additional biological factors may have influenced treatment sensitivity.

An additional layer of complexity emerged from the molecularly informed diagnostic reassessment. The coexistence of a UV mutational signature and a truncating *PTCH1* alteration prompted re-evaluation of the original diagnosis. Integration of histopathologic and immunohistochemical findings with the UV mutational signature and truncating PTCH1 alteration ultimately supported reclassification as basal cell carcinoma with squamous differentiation rather than conventional cSCC, consistent with the recognized clinicopathologic and molecular overlap of basosquamous tumors, in which Hedgehog-pathway alterations, including PTCH1 and SMO mutations, have been reported (17–21). This distinction is clinically consequential as the biological interpretation of the TMB is tumor-type dependent; the same quantitative threshold may carry different functional implications across malignancies driven by distinct oncogenic programs. Here CGP served not only as a therapeutic guide but also as a tool for diagnostic refinement. The case therefore emphasizes that biomarker assessment should not be decoupled from the histologic and molecular context.

The identification of a truncating PTCH1 alteration provided a biologically coherent framework for considering Hedgehog signaling as a potential contributor to the limited objective respons. PTCH1 loss drives constitutive activation of the Hh signaling pathway, a central driver of basal cell carcinoma pathogenesis (18, 19, 22). Beyond its role in tumor cell proliferation increasing evidence suggests that Hh signaling contributes to immune modulation within the tumor microenvironment.

The Hedgehog signaling pathway plays a central role in embryonic development and tissue patterning and is typically quiescent in adult tissues. In basal cell carcinoma, inactivating mutations in PTCH1 release constitutive suppression of Smoothened (SMO), leading to persistent activation of downstream GLI transcription factors and uncontrolled cellular proliferation (18, 19). Vismodegib, a small-molecule SMO inhibitor, blocks this aberrant signaling cascade and is approved for advanced basal cell carcinoma.

Preclinical and translational studies have demonstrated that aberrant GLI activity can promote stromal expansion, reshape cytokine and chemokine networks, and impair cytotoxic T-cell recruitment (23–25). These effects are consistent with the development of an immune-excluded phenotype, characterized by spatial restriction of effector lymphocytes despite preserved antigenicity. Importantly, such immune exclusion may occur independently of mutational burden, suggesting that oncogenic signaling can actively override the immunogenic potential generated by ultraviolet-induced hypermutation. Although direct clinical evidence linking Hedgehog activation to checkpoint inhibitor resistance in basal cell carcinoma remains limited, mechanistic parallels across GLI-driven malignancies support the plausibility of this interaction. In this framework, pathway activation functions not merely as a proliferative driver but as a dominant regulator of immune accessibility.

This distinction between antigenicity and immune accessibility is central to understanding the paradox observed in this patient. High TMB reflects the theoretical availability of neoantigens; however, effective immune engagement requires functional infiltration and activation of cytotoxic effector cells (26, 27). In tumors in which stromal architecture, chemokine gradients, or oncogenic signaling impose structural and immunologic constraints, mutational load alone may be insufficient to drive durable response.

Within this context, the decision to combine Hedgehog pathway inhibition with PD-1 blockade was grounded in a biology-driven hypothesis.

Suppression of GLI-mediated signaling has been associated with normalization of cancer-associated fibroblasts, reduction of immunosuppressive mediators, and enhanced T-cell infiltration in preclinical systems (28, 29). Accordingly, Hedgehog inhibition may function as a microenvironmental modulator capable of converting an immune-excluded tumor into a more permissive immunologic state. In this case, the strategy was not intended to generate additional antigenicity but to restore the functional relevance of an already substantial neoantigen burden.

The clinical course supports this interpretation. During nivolumab plus ipilimumab, the best observed response was stable disease without objective regression. A marked metabolic response was subsequently documented after pembrolizumab and vismodegib were initiated concurrently. Because both agents were introduced simultaneously and the patient had previously received checkpoint blockade, the relative contributions of direct vismodegib activity, pembrolizumab activity, a delayed or carry-over effect of prior immunotherapy, and a possible interaction between the agents cannot be distinguished. The temporal sequence is clinically informative and hypothesis-generating but does not establish agent-specific attribution or therapeutic synergy.

Emerging clinical experience has begun to explore the combination of Hedgehog pathway inhibition with PD-1 blockade in advanced basal cell carcinoma, with early reports suggesting that vismodegib may enhance the activity of pembrolizumab in selected patients (30, 31). Although data remain limited and largely exploratory, these findings provide a clinical context for the strategy employed in the present case and support further investigation of pathway-modulated immunotherapy combinations.The following liquid biopsy findings further inform the biological interpretation. The same PTCH1 alteration remained detectable in plasma during longitudinal assessment. Simultaneously, the maintenance of high TMB argues against neoantigen loss as a mechanism of initial resistance. Together, these observations support a model in which oncogenic signaling constrained immune effectiveness despite high antigenic potential, and in which pathway inhibition shifted this balance.

More broadly, this case contributes to the evolving discussion regarding composite biomarker strategies in immuno-oncology. PD-L1 expression, microsatellite stability, and TMB each capture discrete aspects of tumor–immune interaction, yet none fully account for the influence of tumor-intrinsic signaling networks. Pathways such as Hedgehog, WNT/β-catenin, and PI3K/AKT have increasingly been implicated in shaping immune architecture and therapeutic responsiveness (32). Integrating pathway-level information with conventional immunotherapy biomarkers may provide a more biologically coherent approach to treatment selection, particularly in tumors demonstrating discordant biomarker profiles.

Several limitations merit consideration. As a single case report, causality cannot be established, and the relative contribution of vismodegib versus pembrolizumab remains uncertain. Detailed immune microenvironment characterization was not performed, limiting mechanistic confirmation. Furthermore, prospective validation would be necessary to determine whether the observed interaction between Hedgehog inhibition and PD-1 blockade is reproducible across a broader population. Representative H&E and immunohistochemical images could not be included because the diagnostic reassessments were performed at external laboratories in 2022, and the original slides and corresponding digital images were not transferred to or archived at our institution.

Nevertheless, this case highlights an important principle: extreme TMB does not inherently confer immunologic dominance. When tumor-intrinsic signaling pathways impose immune exclusion, mutational load alone may be insufficient to drive therapeutic benefit. A context-aware interpretation of genomic findings—integrating tumor lineage, oncogenic pathway activation, and immune biomarkers—may enable more rational, biology-guided combinatorial strategies in patients with apparent biomarker discordance.

## Conclusion

This case underscores that ultra-high tumor mutational burden, even in the presence of a dominant UV mutational signature, does not automatically translate into meaningful benefit from immune checkpoint blockade. When tumor-intrinsic oncogenic pathways impose immune exclusion, mutational load alone may be insufficient to drive therapeutic benefit.

The detection of a truncating *PTCH1* alteration was pivotal. It prompted diagnostic reassessment and redirected treatment toward Hedgehog pathway inhibition. The durable response observed after combining vismodegib with PD-1 blockade suggests that pathway activation may have played a central role in shaping immune resistance.

Rather than interpreting TMB in isolation, this case highlights the importance of integrating molecular drivers, tumor lineage, and immune biomarkers in therapeutic decision-making. In selected patients, targeting oncogenic pathways alongside immunotherapy may help address apparent resistance despite high mutational burden.

## Data Availability

The original contributions presented in the study are included in the article/supplementary material, further inquiries can be directed to the corresponding author/s.
